# Life Cycle Assessment of the Separation and Recycling of Fluorinated Gases Using Ionic Liquids in a Circular Economy Framework

**DOI:** 10.1021/acssuschemeng.1c04723

**Published:** 2021-12-17

**Authors:** Daniel Jovell, Josep O. Pou, Fèlix Llovell, Rafael Gonzalez-Olmos

**Affiliations:** † Department of Chemical Engineering and Materials Science, IQS School of Engineering, Universitat Ramon Llull, Via Augusta 390, 08017 Barcelona, Spain; ‡ Department of Chemical Engineering, Universitat Rovira i Virgili, Avinguda Països Catalans 26, 43007 Tarragona, Spain

**Keywords:** Fluorinated gases recovery, Ionic liquids, COSMO-RS, Life cycle assessment (LCA), Circular economy

## Abstract

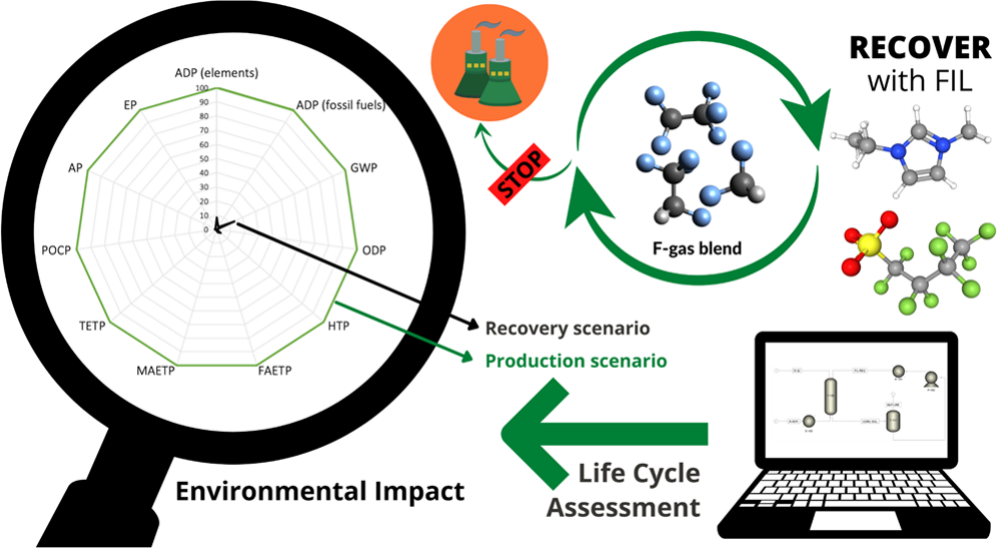

The stricter regulation regarding the use of fluorinated gases (F-gases), as a consequence of their high Global Warming Potential (GWP), represents a challenge for the refrigeration industry. The design of alternatives requires the recycling of the low to moderate GWP compounds from current refrigerant blends. However, there is not a developed and standardized technology available to recover them, and once the life cycle of the refrigeration equipment has ended, most gases are incinerated. Fluorinated ionic liquids (FILs) can effectively perform as absorbents to the complex separation of F-gas mixtures. In this work, a methodology based on the COSMO-RS thermodynamic package integrated into an Aspen Plus process simulator was used to evaluate the performance of an FIL to recover difluoromethane (R-32) from the commercial blend R-407F. The environmental sustainability of the recovery process (circular economy scenario) was analyzed with a life cycle assessment (LCA) approach, comparing the obtained results with the conventional R-32 production (benchmark scenario). The results reveal a 30% recovery of 98 wt % R-32 suitable for further reuse with environmental load reduction in the 86–99% range compared to the R-32 production. This study can guide the development of new F-gas recovery technologies to improve the environmental impacts of these compounds from a circular economy perspective.

## Abstract 2

Recovery of low-GWP F-gases with ionic liquids in circular economy scenarios can achieve environmental impact reductions higher than 86%.

## Introduction

Within the context of the battle against climate change, the impact of fluorinated greenhouse gases (F-gases), and particularly of the hydrofluorocarbons (HFCs) used in the refrigeration industry, is one of the major short-term concerns. These gases are capable of reaching a global warming potential (GWP) thousands of times the CO_2_ value and have a long atmospheric lifetime. The contribution of F-gases is projected to triple from nearly 2% to around 6% of greenhouse gas emissions by 2050.^1^ On this account, the Kigali’s amendment to the Montreal Protocol, entered into force in 2019, includes a global compromise to limit HFCs refrigerants’ production to reduce the expected half a degree temperature increase due to F-gases by 2100. In this direction, the 517/2014 regulation of the European Parliament has strict targets by 2030, including a reduction of 79% HFC consumption and 67% HFC emissions, using the 2009–2012 period as the baseline.^2^ To achieve these goals, a progressive phase-down of high GWP F-gases has been set up by prohibiting the use of nonrecycled high GWP compounds, as well as establishing yearly decreasing quotas of produced and imported HFCs. Additional regulatory rules include a rigorous control of the equipment maintenance and of the F-gas recovery, recycle, or destruction at the equipment end-of-life (EoL) to avoid fugitive emissions.^3^ This strict regulation is pushing the market to find innovative solutions, as many current commercial blends, such as R-410A (GWP = 2255.5) and R-407F (GWP = 1965.3), which include at least one high GWP refrigerant (pentafluoroethane, R-125, GWP= 3740),^4^ will be phased out in the coming years.

While a midterm solution consists of the replacement of these mixtures by other systems with a lower environmental impact, a major issue of the F-gas industry is the waste generation and the lack of effective treatments for the recycling of these gases. Indeed, most refrigeration blends are removed from the cooling circuit at the EoL of the equipment and transported to be incinerated.^3^ While incineration stands as an effective process for the destruction of HFCs, the release of CO_2_ and generation of byproducts, such as trifluoroacetate, may cause atmospheric and ecosystem damage.^3^

The separation of azeotropic mixtures of HFCs forming current refrigeration blends, for further reuse of the individual compounds, is a difficult task. Consequently, it is essential to explore new key-enabling technologies (KETs) for the sustainable recovery of F-gases to evolve toward a context of circular economy, where the recovered HFCs can be used to formulate new refrigerant blends, mixed with either hydrofluoroolefins (HFO)^5−8^ or hydrofluoroethers (HFE),^9−11^ with lower GWP and null ozone depleting potential (ODP).

Recent publications have explored advanced separation processes such as absorption with ionic liquids (ILs),^12−15^ selective adsorption (with activated carbons,^16,17^ zeolites,^18^ and metal organic frameworks),^19^ and membrane separation.^20−22^ Regarding the use of selective absorption processes, ILs have gained attention for their particular properties: a near-zero vapor pressure, wide liquid range, nonflammability, and, more importantly, the capability of tuning the physicochemical properties by changing the structure of the absorbent, with several publications addressing the feasibility of the selective absorption of HFC blends.^12−15^ Given this fine-tuning capacity, fluorinated ionic liquids (FILs) and deep eutectic solvents^14^ have been recently studied as absorbents for fluorinated compounds such as HFCs.^23^ FILs are composed of fluorinated alkyl chains with a minimum of four carbon atoms in their chemical structure. The combination of fluorines and carbons, along with the typical ionic liquid cation–anion interactions, results in the formation of fluorinated, polar, and nonpolar nanosegregated areas within the same molecule, increasing their number of conformations and their capacity to absorb F-gases.^24,25^ Among the recent studies, Sosa et al.^26^ reported remarkable solubilities of difluoromethane, R-32; 1,1,1,2-tetrafluoroethane, R-134a; and R-125 in FILs. On the basis of the Henry’s constants provided in this work, the choice of 1-ethyl-3-methylimidazolium perfluoropentanoate, [C_2_mim][C_4_F_9_CO_2_], seems to be the best option to separate R-32 in a single absorption column, as its IL affinity is significantly lower than R-134a and R-125. This experimental evidence is of particular interest for the case of the high GWP R-407F blend (a ternary mixture of 30 wt % of R-32, 30 wt % of R-125, and 40 wt % of R-134a) where R-32 has a low GWP and can be recycled for further use.

In spite of all these recent efforts toward the development of separation technologies, there is no information on the environmental cost of these alternatives. In this regard, it is necessary to ensure, through a careful life cycle analysis (LCA), the possible benefits of these new separation units, quantifying these environmental benefits with respect to the impacts generated during the production of fresh F-gas.

LCA represents a complete tool that takes into account several categories of environmental impact during the life cycle of products and services. Several studies have been devoted to CO_2_ capture for the reduction of the emissions of this gas, with recent work on a comprehensive guideline for the LCA of carbon capture and utilization (CCU) technologies.^27^ Within this framework, an in-depth analysis, based on the LCA methodology, of the F-gas capture technology is a necessary tool to provide a complete picture of these greenhouse gas mitigation strategies from a circular economy context. To our knowledge, no work has been published about the environmental impact of F-gas recovery technologies.

Although the idea of gas waste recycling seems to be, a priori, beneficial, the use of ILs in the F-gas recovery requires a deep study of its environmental impacts using a life cycle approach,^28−30^ with particular attention to the shortcomings that may occur due to the lack of information on these compounds.^29^ It is then essential to perform an LCA study to compare the F-gas recovery versus the conventional F-gas production.

Consequently, this contribution intends to assess the sustainability of a novel absorption FIL-based process using [C_2_mim][C_4_F_9_CO_2_] to separate and recover R-32 from R-407F using a holistic approach from a computational perspective, including a rigorous thermodynamic molecular study, a detailed process simulation to optimize the recovery process, and an LCA study of the R-32 recovery process compared to their current linear production and destruction after use.

## Materials and Methods

### Property Model Specification and Component Definition in Aspen Plus

The absorption process has been modeled in Aspen Plus v11 (37.0.0.395) using the COSMO-SAC (conductor-like screening model with segment activity coefficient) property model. COSMO-SAC is a solvation model for polarizable species that depicts the electric fields on the molecular surface.^31^ Individual atoms, rather than functional groups, are used as building blocks in this activity-coefficient model, increasing the range of applicability without relying on experimental evidence for binary interaction parameters. This particular feature makes the COSMO-SAC method especially attractive for cases where the species are not defined in the simulator database, such as the ionic liquid used in this work.^31^

The thermodynamic model requires rather complex quantum mechanical calculations for each component. This includes the COSMO volume, CSACVL, as well as its charge distribution profile (Figure S1 and Table S1), which may store up to 12 σ-profile points. All six parameters are obtained using COSMO-RS calculations. Further computational details are included in the Supporting Information (Section A1).

R-32, R-125, and R-134a F-gases forming the R-407F blend are included as conventional components from the Aspen Plus database, whereas the FIL [C_2_mim][C_4_F_9_CO_2_] is incorporated as a pseudocomponent. To do so, molecular weight, normal boiling temperature (NBP), and density at 60 °F are specified. The remaining properties required to fully specify the component are obtained from property estimation using the API procedures with Aspen’s modifications.

In addition to the properties retrieved from API procedures, several scalar and temperature-dependent properties were included in the properties specification environment to make the simulation thermodynamically consistent. The boiling point, critical temperature, critical pressure, critical compressibility factor, and acentric factor were all calculated using Valderrama and co-workers’ modified Lydersen–Joback–Reid group contribution method.^32^ The temperature-dependent properties include heat capacity, viscosity, and density of the selected FIL. The heat capacity was estimated from Valderrama’s group contribution method^33^ based on the mass connectivity index,^34^ while the viscosity and the density were retrieved from available experimental data.^35^ The measurements were implemented in Aspen Plus and regressed in the desired range of temperatures and pressures. Data regression is based on the Least Absolute Residuals (LAR) method together with the Levenberg–Marquardt algorithm. The negligible vapor pressure of the FIL has also been taken into account by setting the extended Antoine equation implemented in Aspen plus (PLXANT) to an imaginary value of −1 × 10^18^.^36^ Both the scalar properties and the coefficients for each correlated property model are available in Tables S2 and S3 of the Supporting Information, respectively.

### Fluorinated Gas Absorption Process

The flow diagram of the absorption process studied in this work is depicted in the Supporting Information (Figure S2). It consists of an absorption column for the separation of R-32 from the original R-407F blend using [C_2_mim][C_4_F_9_CO_2_] as an absorbent, followed by a flash tank for the regeneration and recycling of the FIL. The recirculation stream includes a pump (P-100) and a heat exchanger to cool down the FIL before returning to the main column. R-407F is assumed to arrive bottled at 8 bar and ambient temperature (25 °C) and is heated up to 45 °C (E-100) and fed counter-currently at a rate of 1000 kg/h in the absorption column (T-100) with the FIL at 14 °C and 8 bar.

R-125 and R-134a are selectively absorbed, and an enriched R-32 gas stream is released from the top of the absorber. The FIL solution with the absorbed R-125 and R-134a is fed into the flash drum (T-101). The FIL is then regenerated by a pressure swing (i.e., reducing the pressure to 0.01 bar) and cooled down to 14 °C (E-101) before it is recycled back to the absorption column for further reuse. The desorbed gases are released in the vapor stream of the flash drum.

The absorber has been modeled using the RadFrac module available in Aspen Plus. The equilibrium approach, in which the liquid and vapor phases are assumed to be in equilibrium, is chosen together with the petroleum/wide-boiling convergence algorithm. A purity of 98 wt % for R-32 is set as a design specification, using the FIL rate as a manipulated variable, to ensure an adequate performance of the recycled refrigerant.

The utilities used in the simulation for the duties of the pump and the two heat exchangers were electricity (pump P-100 and heater E-101) and low-pressure steam (heater E-100). Pump and driver efficiencies of 0.65 and 0.85 were considered, respectively.^23^

### Life Cycle Assessment

The life cycle assessment study follows the methodology proposed by Wu et al.^28^ The cradle-to-gate life cycle environmental impacts of the R-32 recovery (circular economy scenario) with FIL from the R-407F mixture and the R-32 industrial production (Benchmark scenario) were calculated with the software SimaPro (version 9.0). The LCA is carried out following the steps reported in the ISO 14040:2006 standard: (1) goal and scope definition, (2) life cycle inventory (LCI) analysis, (3) life cycle impact assessment (LCIA), and (4) results interpretation.^37^

**Figure 1:**
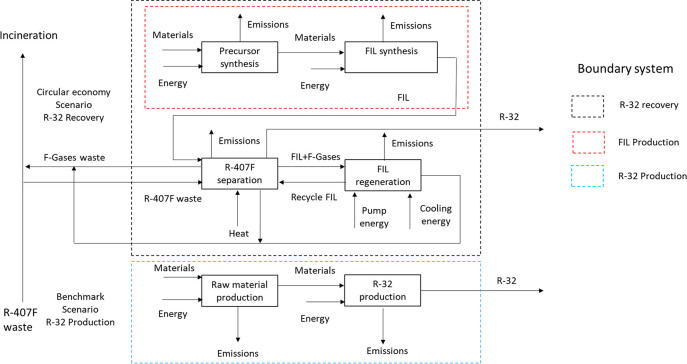
LCA system boundary of the R-32 recovery and production scenarios. The system boundary includes transportation processes, although they are omitted in the diagram to facilitate the diagram interpretation.

The main goal of our article is to build an integrated LCA framework, comparing the life cycle environmental impacts among the recovery of the R-32 from used refrigerant mixtures using the FIL [C_2_mim][C_4_F_9_CO_2_] and the R-32 industrial production. The scope of this study comprises: (1) the construction of the life cycle framework and LCI analysis for the two scenarios (R-32 recovery vs R-32 production); (2) a comparison of the LCIA of the studied scenarios; (3) an uncertainty analysis using Monte Carlo methodology to verify the robustness of the obtained results; and (4) a sensitivity analysis to recognize the most important factors that affect to the environmental impacts.^28^ The boundary of the system is shown in Figure 1 and includes all the processes of the R-32 recovery and R-32 production scenarios. It also includes transportation processes, which are omitted in the diagram to facilitate the interpretation. The process proposed is considered a closed-loop recycling in which the recovered R-32 has the same quality and value than the produced R-32, so it can be recycled indefinitely without losing quality or functional (cooling) properties. On the basis of this assumption, allocation procedures for the burdens associated with avoiding the production of fresh R-32 and its incineration were not considered to simplify the LCA.

The LCI of the R-32 recovery contains all the inputs and outputs (mass and energy), and the emissions associated with the precursors production, FIL production, F-gases separation, and FIL regeneration. For precursors and FIL production, the LCI includes the upstream feedstock, auxiliary materials (i.e., catalyst), and energy consumption (i.e., cooling, heating...). The inventory of R-32 separation comprises the FIL inputs, losses, and energy consumption. The data for thermal energy (natural gas), electricity (electricity mix grid), chemical plants (chemical factory, organics), transportation (freight, lorry 16–32 t), and EoL of the wasted FIL (hazardous waste incineration) are obtained from Ecoinvent 3.^38^ All of the industrial processes are considered to take place in Spain.

The precursors and the used FIL are not available in the LCI databases. The data for these compounds were obtained from the literature or process simulation (PS).^28^ Following the assumptions of published works,^27,28^ it was considered an emission to the air of 0.2% of input materials in those processes without information in Ecoinvent except for the FIL, which is considered to have a negligible vapor pressure. For the precursors’ production, the theoretical energy needed is industrially up-scaled through the use of conversion factors.^28,39^ The theoretical heat needed in endothermic reactions is multiplied by a factor of 4.2, considering that the heat is obtained from natural gas. In the case of exothermic reactions, the theoretical heat generated is multiplied by a factor of 3.2, considering cooling electricity from the Spanish electricity mix.^28^

The LCIA of the R-32 absorption is carried out with the CML-IA methodology,^40^ which has been recently suggested for studies with ILs.^29^ The following environmental impact categories were studied in the LCIA (further details are given in Supporting Information section A2): global warming potential (GWP; kg CO_2_ equiv/kg emission), abiotic depletion of elements, ultimate reserves (ADP elements; kg SB equiv/kg extraction), abiotic depletion of fossil fuels (ADP fossil fuels; MJ/kg), ozone depletion potential (ODP; kg CFC-11 equiv/kg emission), human toxicity potential (HTP; kg 1,4-dichlorobenzene equiv/kg emission), freshwater aquatic ecotoxicity potential (FAETP; kg 1,4-dichlorobenzene equiv/kg emission), marine aquatic ecotoxicology potential (MAETP; kg 1,4-dichlorobenzene equiv/kg emission), terrestrial ecotoxicity potential (TETP; kg 1,4-dichlorobenzene equiv/kg emission), photochemical oxidation potential (POCP; kg ethylene equiv/kg emission), acidification potential (AP; kg SO_2_ equiv/kg emission), and eutrophication potential (EP; kg PO_4_ equiv/kg emission). Normalization using European normalization factors (EU25) was used to convert the characterization results of all impact categories into dimensionless scores.

## Results and Discussion

### Thermodynamic Validation

**Figure 2:**
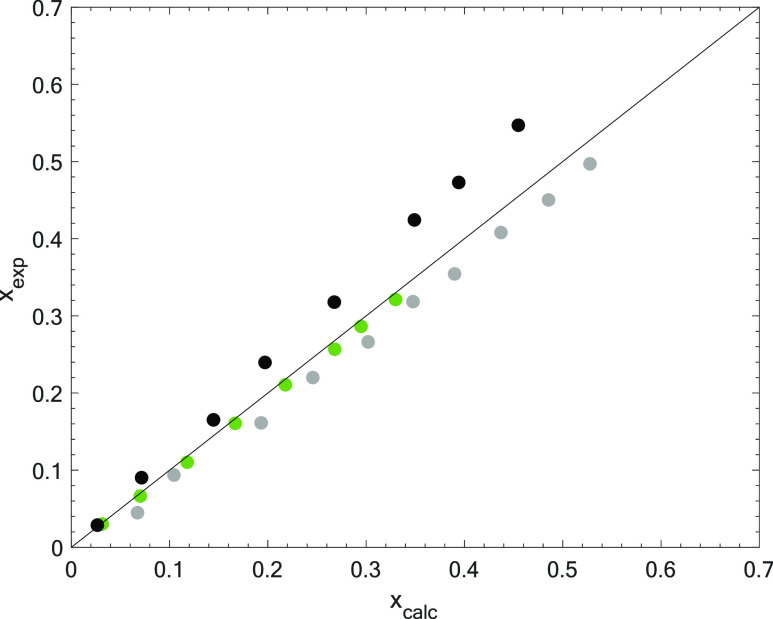
Validation of F-gas solubility in [C_2_mim][C_4_F_9_CO_2_] prediction from the COSMO-RS-based simulation approach with experimental data from Sosa et al.^26^ for R-32 (green), R-125 (black), and R-134a (gray) at 303.15 K.

COSMO-RS-based simulation results were validated by comparing experimental solubility measurements^26^ with those calculated with Aspen Plus. The comparison was made for all three fluorinated gases forming R-407F by simulating a simple equilibrium flash model and setting the desired output pressure (up to 6 bar given the available experimental data).^26^ It should be noted that COSMO-RS calculations are directly performed in Aspen Plus to obtain the desired properties (see section A1 from the Supporting Information). As it can be seen from Figure 2, COSMO-based Aspen predictions are capable of faithfully reproducing the gas solubility in the selected FIL and give acceptable deviations in terms of the percentage of absolute average deviation (R-32 = 4.09%, R-125 = 18.79%, and R-134a = 12.00%) in the whole range of pressures for which experimental data have been reported. Although the prediction worsens with the length of the alkyl chain and the pressure, these results have comparable accuracy to those published by other authors in similar studies where the solubility of gases in ILs was predicted with COSMO-RS.^23,41,42^

### R-32 Separation Process with [C_2_mim][C_4_F_9_CO_2_] FIL

The main results for the Aspen Plus simulation are presented in Table 1. The results show that a column with 38 theoretical stages, the minimum number in which the module converged, is necessary to achieve an R-32 purity of 98 wt %.

**Table 1:** Main Results of the ASPEN Plus Simulation for the Separation of R-32 from the R-407F Blend

R-407F feed	1000.00	kg/h
R-32 separated (98 wt %)	90.90	kg/h
fluorinated ionic liquid	5774.07	kg/h
R-32 recovery	30.3	%
ratio L/G	5.77	kg FIL/kg R-407F
annual R-32 production	719.94	tonnes of R-32
absorber (T-100) theoretical stages	38	
absorber (T-100) operating pressure	8	bar
cooler electrical consumption	7.03	kW
heater consumption	4.64	kW
pump electrical consumption	1.56	kW

A working pressure of 8 bar has been fixed as a compromise between an acceptable R-32 recovery ratio and a low energy consumption. Overall, the energy consumption of the process is modest (see Table 1) given the relatively low operation flows and the moderate temperature operation range. Further insight will be given in this aspect in the LCA analysis.

A 30.3% recovery of R-32 is achieved using 5774 kg/h of FIL [C_2_mim][C_4_F_9_CO_2_], which is also recovered and recycled. The recovery of R-32 obtained, in a similar L/G ratio and pressure conditions, is in the same range as those published by other authors for diluted mixtures of R-32 in argon using similar FILs.^23^ Sensibly lower values of the L/G ratio are obtained (5.77) compared to similar absorption separation processes involving imidazolium-based ionic liquids for the capture of CO_2_ and tetrafluoroethylene (11.7–70.0).^36,41^ The results corroborate the adequacy of the FIL selected and provide, for the first time, a technology process for the separation of the R-32 from a ternary mixture in an efficient-sustainable way.

### Material and Energy Flow Analysis

**Figure 3:**
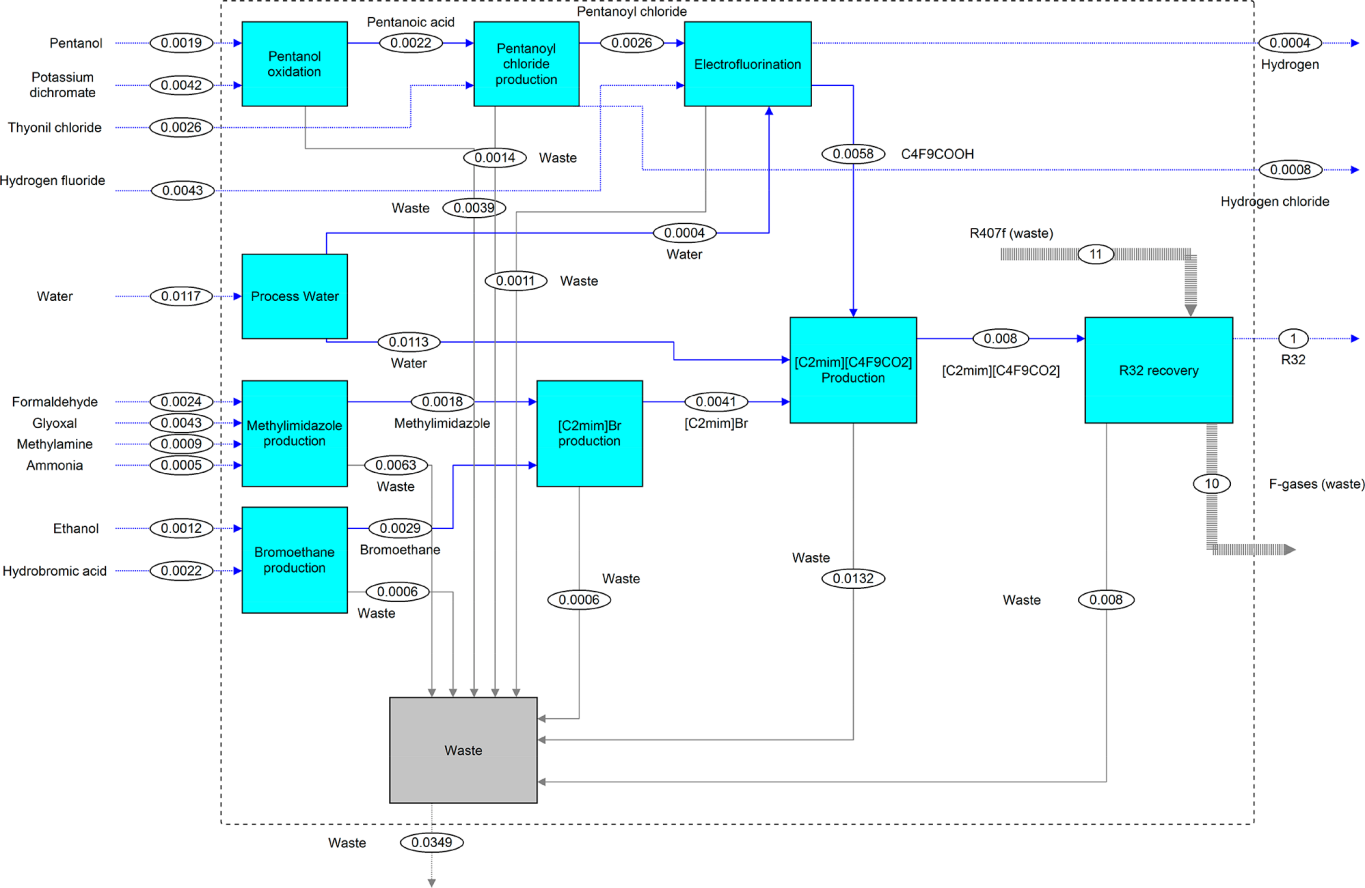
MFA for the recovery of 1 kg of R-32 using [C_2_mim][C_4_F_9_CO_2_] as an absorbent. Units: kilogram. The thickness of the line shows the relative material flow.

In order to understand the use of materials and their transformation during R-32 recovery, a material flow analysis (MFA) is carried out considering the separation of 1 kg of R-32 from the R-407F blend with a purity higher than 98 wt %. Figure 3 shows the results obtained from the MFA. According to the results of the previous simulated process, the FIL is completely regenerated for further reuse. However, it is possible that, after prolonged use, the FIL may lose separation efficiency. For that reason, it has been established that the FIL will be replaced after one year (considering a total FIL mass in the close circuit of 5774 kg). The effect of this parameter on the LCA will be assessed in the sensitivity analysis section. On the basis of this assumption and the simulation results, 0.008 kg of [C_2_mim][C_4_F_9_CO_2_] is needed to separate 1 kg of R-32. For the production of 0.008 kg of [C_2_mim][C_4_F_9_CO_2_], 0.036 kg of raw materials is needed (derived from the production of the cation and anion precursors, [C_2_mim]Br and C_4_F_9_COOH, respectively). The material flow data for the [C_2_mim]Br production was obtained from published data,^28^ while C_4_F_9_COOH production material flow data was estimated through PS based on the process reported by Kauck et al.^43^ The mass of the used FIL ([C_2_mim][C_4_F_9_CO_2_]) is 22.2 wt % of the total raw materials used in the precursors’ production. The total amount of waste emission in the [C_2_mim][C_4_F_9_CO_2_] production process is 0.013 kg, accounting for 36.4 wt % of the total raw materials. It is important to highlight the use of water during the FIL production, accounting for 33.5 wt % of the emissions related to the FIL production. In the FIL use stage, 0.008 kg of waste related to the FIL replacement is produced, accounting for 22.9 wt % of the total waste emission. Hydrogen chloride and hydrogen are byproducts produced during the production of pentanoyl chloride and C_4_F_9_COOH, respectively, which can be considered as “avoided products”.^28^ These byproducts stand for 3.2 wt % of the raw materials introduced in the system.

**Figure 4:**
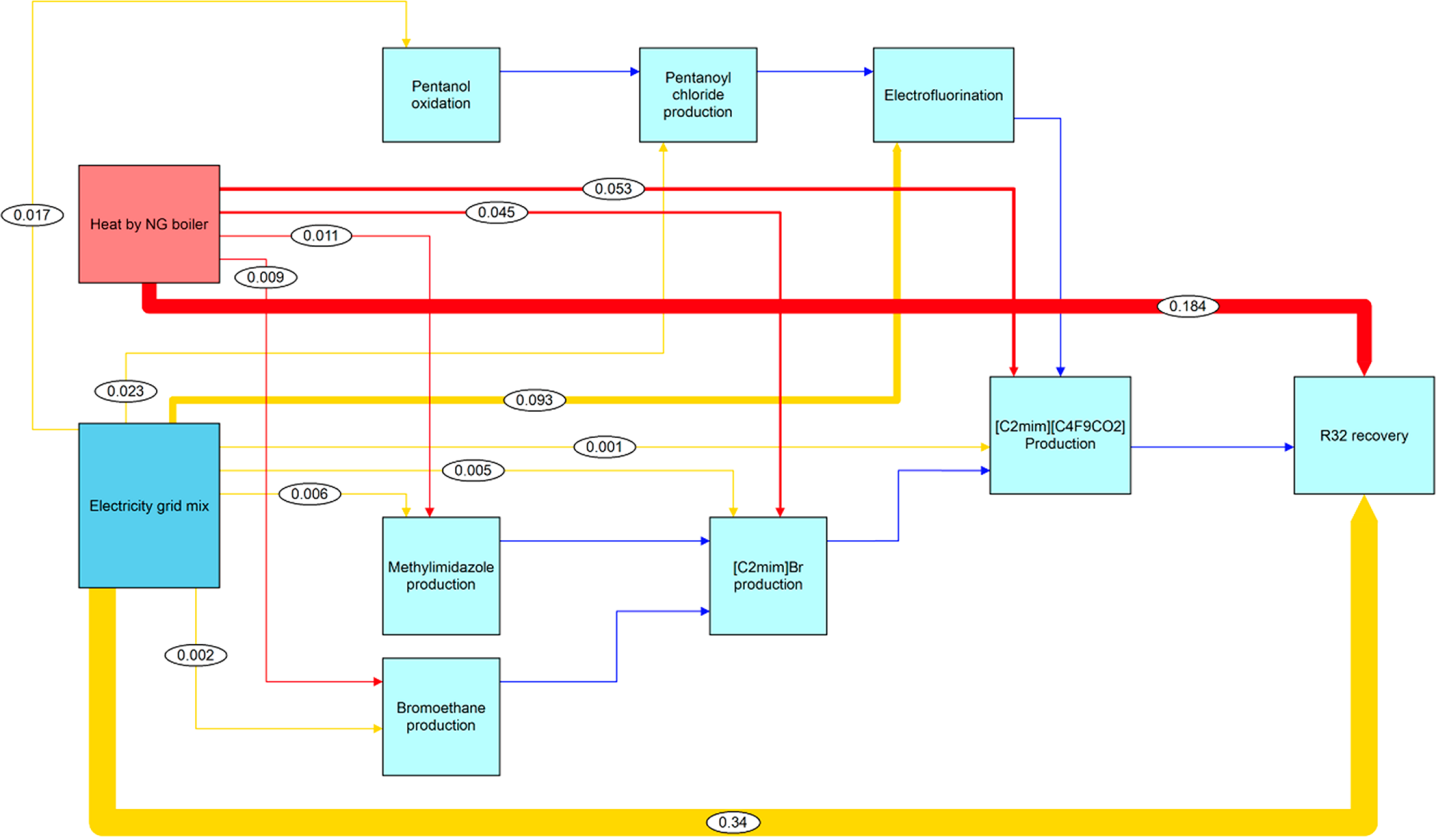
EFA for the recovery of 1 kg of R-32 using [C_2_mim][C_4_F_9_CO_2_]. Red arrows represent heat (MJ_th_), while yellow arrows represent electricity (MJ_el_). The thickness of the line shows the relative energy flow.

Figure 4 shows the energy flow analysis (EFA) of the recovery of 1 kg of R-32 considering the electricity and heat consumption (e.g., mixing, heating, pressurization, cooling) during the production of precursors and FIL and R-32 recovery. The energy consumption inventory in the [C_2_mim]Br production is obtained from published studies.^28^ The energy consumption data of the C_4_F_9_COOH production, FIL synthesis, and the R-32 recovery process are obtained with the PS carried out in this study (Table S4).^35,43,44^ The results of the EFA show that the electricity consumption was 69.8% in the R-32 recovery, 30.0% in the FIL precursor production, and 0.2% in the FIL production. In the case of heat consumption, a similar distribution was observed (60.9% was in the R-32 recovery, 21.6 in the FIL precursors production, and 17.5% in the FIL production). From the EFA results, it is clear that the decrease of the electricity and heat consumption during the R-32 recovery is a promising strategy to reduce the total life cycle energy consumption.

### Environmental Impacts Analysis of R-32 Recovery

**Figure 5:**
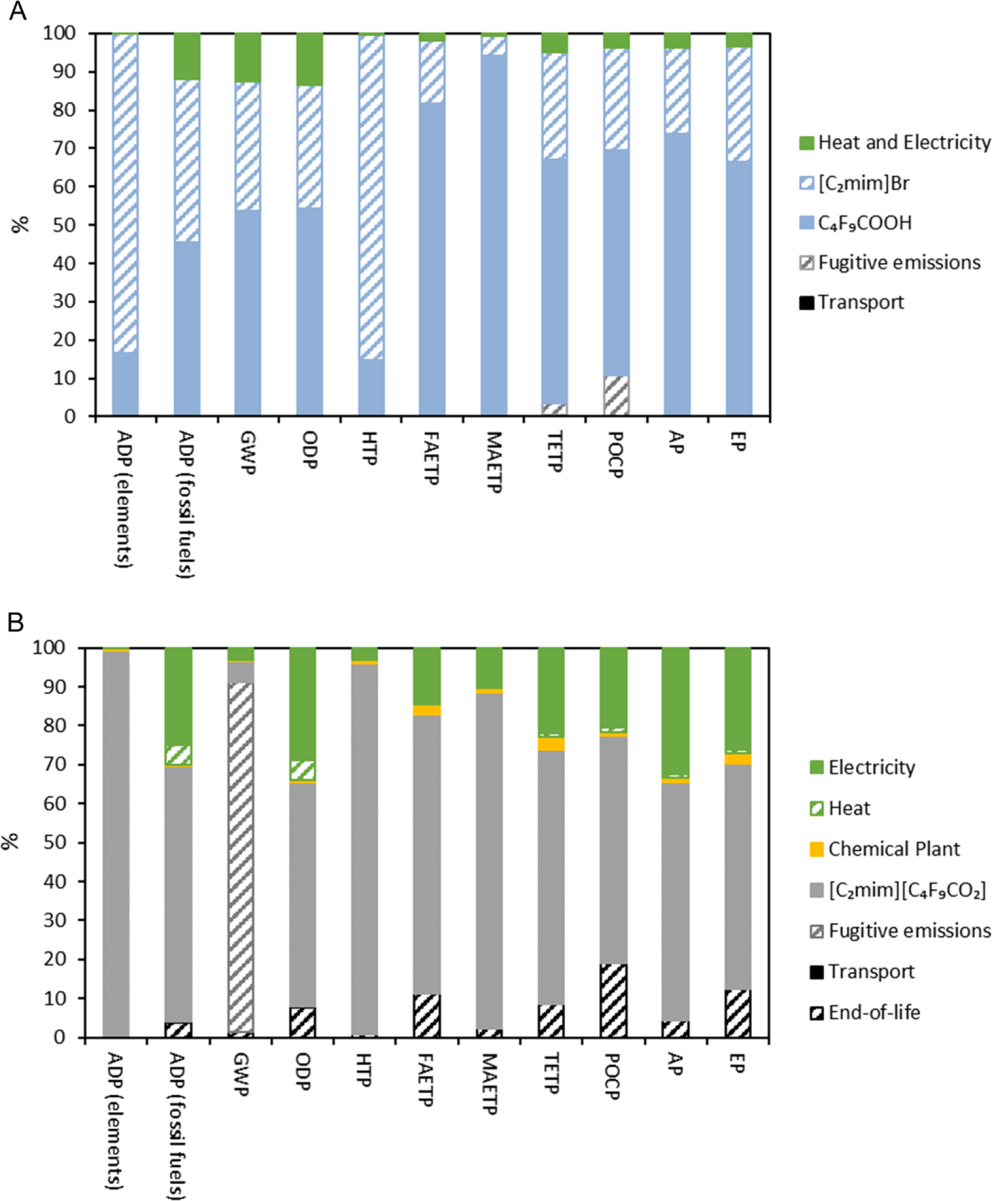
Characterization of the environmental impacts of (A) 1 kg of [C_2_mim][C_4_F_9_CO_2_] production and (B) recovery of 1 kg of R-32 from R-407F with [C_2_mim][C_4_F_9_CO_2_].

First, the distribution of the environmental impacts of the absorbent production (1 kg of FIL [C_2_mim][C_4_F_9_CO_2_]) in the different categories is provided in Figure 5A. The results show that the production of [C_2_mim]Br and C_4_F_9_COOH has the largest contribution in all of the impact categories (contributions between 85 and 100%). Moreover, C_4_F_9_COOH has the most important contribution in all of the categories excluding ADP (elements) (16.5%) and HTP (14.9%). The impact category with the highest contribution of C_4_F_9_COOH is MAETP (94.3%), caused mainly by the emission of hydrogen fluoride. [C_2_mim]Br also has a severe contribution to the impact categories of the FIL production, ranging from 5 to 85%, depending on the category. [C_2_mim]Br has the most relevant contributions to the HTP (85.9%) and ADP (elements; 83.4%) categories. As previously reported by Wu et al.,^28^ the high contribution in the HTP category is attributed to the ethylene oxide emissions, which are considered to be toxic to humans. The contribution in the ADP (elements) is mainly caused by the extraction of bromine and chromium. However, the process energy (electricity and heat) accounts for the environmental impact in the range 0–14% depending on the impact category. In the production of [C_2_mim][C_4_F_9_CO_2_], the transportation processes only account for 0.01–0.72% of the environmental impacts. Consequently, compared to the rest of the processes, the contribution of transportation to the life cycle impacts of the [C_2_mim][C_4_F_9_CO_2_] production can be omitted. The LCIA results of the FIL production process are included in the Supporting Information (Table S5).

Second, the environmental impacts of the recovery process of 1 kg of R-32 using [C_2_mim][C_4_F_9_CO_2_] as an absorbent from wasted R-407F are estimated. The detailed contributions can be observed in Figure 5B. The results indicate that the use of the FIL in the separation process has the most significant contribution (58–99%) in most of the impact categories except in GWP (5.4%). It is worth mentioning that in the GWP category, 90% of the emissions are caused by the estimated fugitive emissions. The energy consumption (used for pumping, heating, cooling down, and regenerating the FIL) contributes to 0.11–34% of the environmental impact categories. The EoL of the wasted FIL contributes to 0.10–19% of the environmental impacts. Finally, the construction of the chemical plant facility contributes less than 3% to the environmental impact categories, and as observed during the FIL production, the transportation contribution to the environmental impacts is also residual (0.01–0.72%).

### LCA Comparison Results of R-32 Recovery with Benchmark Scenario

The benchmark scenario for comparative purposes is the current scenario of R-32 production. The LCA of the production of R-32 was carried out according to the information obtained from the patent of Yuichi et al.,^45^ where R-32 is produced from the reaction of dichloromethane with hydrogen fluoride and the available information in Ecoinvent for the production of the fluorinated gas 1,1-difluoroethane, R-152a. The amount of raw materials needed was calculated through the stoichiometry of the reaction obtained from the patent, while the energy and ancillary materials consumption were obtained from the Ecoinvent database, assuming that R-152a and R-32 are relatively similar compounds, due to the lack of available information for R-32. Figure S3 shows the distribution of the impacts to produce 1 kg of R-32. It can be observed how the highest impact in most of the categories, except MAETP and HTP, is caused by hydrogen fluoride and dichloromethane raw materials, falling in the range 15–99% depending on the impact category. The fugitive emissions caused the most important impacts in the HTP and MAETP categories, mainly due to hydrogen fluoride emissions, which is a well-known corrosive and toxic compound. The heat and electricity used in the R-32 production process caused 0.17–12% of the impacts, while the construction of the chemical plant and the transportation had a limited contribution (lower than 3.3% and 0.9% in all impact categories, respectively).

A comparison between the LCAs of the benchmark scenario (R-32 production) and the circular economy scenario (R-32 recovery process) was conducted (Table 2), and the process inventories are shown in Tables S6 and S7, respectively. Comparing the results shown in Table 2, it can be observed that the circular economy recovery scenario has significantly lower environmental loads in all the impact categories than the benchmark production scenario, with a reduction in the range between 86% and 99%. This is caused due to a higher use of raw materials and energy (heat and electricity) in the benchmark scenario compared to the circular economy scenario. The normalized environmental impacts, summarized in Table 2, show how the contribution with the highest impact is the MAETP category in both scenarios. The normalized results also suggest a better environmental performance for the circular economy scenario, which has lower environmental impacts by at least 1 order of magnitude. Figure S4 shows the normalized environmental impact distribution of both scenarios, and it is observed how the highest impact in the MAETP category in the benchmark scenario is caused by the fugitive emissions, while in the circular economy scenario, it is caused by the use of the FIL.

**Table 2:** Characterization and Normalized Life Cycle Impact Results of Production and Recovery of 1 kg of R-32^a^

		benchmark scenario		circular economy scenario	
		R-32 production		R-32 recovery	
impact category	charact. units	charact.^b^	normal^c^	charact.^b^	normal^c^
ADP (elements)	kg Sb equiv	1.39 × 10^–4^	1.64 × 10^–12^	1.17 × 10^–5^	1.38 × 10^–13^
ADP (fossil fuels)	MJ	1.15 × 10^2^	3.65 × 10^–12^	2.13	6.78 × 10^–14^
GWP	kg CO_2_ equiv	1.09 × 10^1^	2.18 × 10^–12^	1.50	2.99 × 10^–13^
ODP	kg CFC-11 equiv	1.16 × 10^–4^	1.30 × 10^–12^	1.92 × 10^–8^	2.15 × 10^–16^
HTP	kg 1,4-DB equiv	9.37	1.21 × 10^–12^	3.62 × 10^–1^	4.67 × 10^–14^
FAETP	kg 1,4-DB equiv	2.17	4.20 × 10^–12^	5.13 × 10^–2^	9.90 × 10^–14^
MAETP	kg 1,4-DB equiv	7.87 × 10^4^	6.74 × 10^–10^	4.91 × 10^2^	4.21 × 10^–12^
TETP	kg 1,4-DB equiv	3.63 × 10^–2^	7.48 × 10^–13^	2.19 × 10^–4^	4.52 × 10^–15^
POCP	kg C_2_H_4_ equiv	3.60 × 10^–3^	4.25 × 10^–13^	5.61 × 10^–5^	6.63 × 10^–15^
AP	kg SO_2_ equiv	9.03 × 10^–2^	3.21 × 10^–12^	8.94 × 10^–4^	3.17 × 10^–14^
EP	kg PO_4_ equiv	1.39 × 10^–2^	1.05 × 10^–12^	2.02 × 10^–4^	1.53 × 10^–14^

a Normalization obtained with European normalization factors (EU25).

b The units of characterization results are indicated in Table 2.

c The normalization results do not have units.

Therefore, the results point out that the environmental benefits of recycling the R-32 instead of producing new fresh R-32 are high. As an example, the GWP of the R-32 production is 10.9 kg CO_2_ equiv, while this value is much lower if the R-32 (1.50 kg CO_2_ equiv) is recovered by the absorption process. According to these results, the carbon footprint of R-32 can be reduced up to 86%, promoting fluorinated gas recovery treatments for their recycling in a circular economy context. Other categories, such as ODP or toxicity, can be reduced by more than 99% and 96%, respectively, in the recovery scenario.

The uncertainty analysis results (see Supporting Information section A3) can give further confirmation of the R-32 recovery LCA results reported in this work from a statistical aspect.

### Sensitivity Analysis

**Figure 6:**
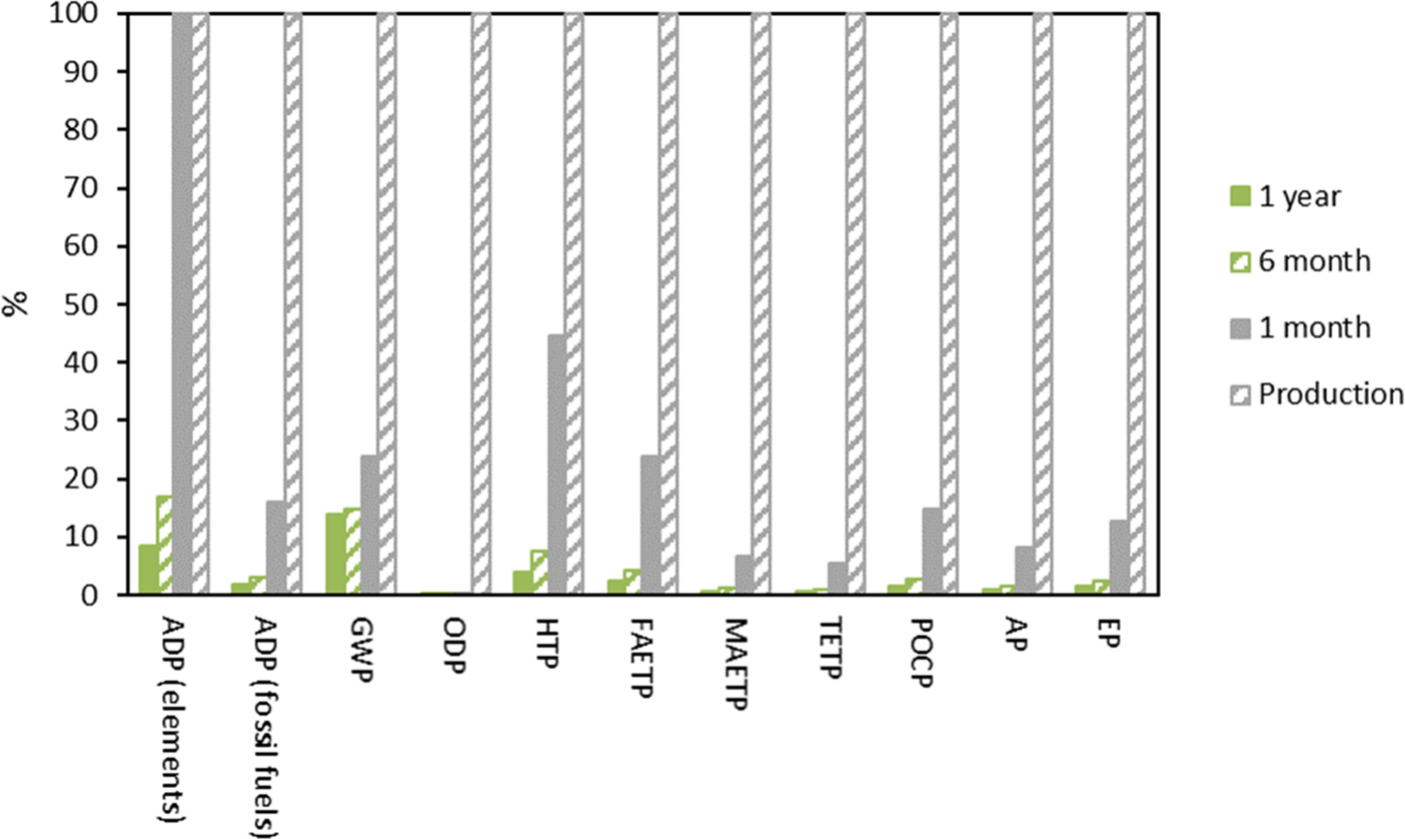
Sensitivity analysis of the replacement frequency of the FIL in the recovery process compared to the benchmark scenario. Impacts referred to the production of 1 kg of R-32.

There is no information in the literature about the recyclability of FILs in HFC absorption processes. Many authors theoretically assumed that, due to its extremely low vapor pressure, FILs can be regenerated in absorption processes without losing absorption efficiency.^12,23^ From experience with other regenerable IL-based absorbents in CO_2_ capture, it is plausible that, with time, the FIL could lose separation efficiency due to degradation processes or interactions with some impurities.^46^ In this work, a replacement of the whole FIL of the closed circuit (5774 kg of FIL) was considered after one year of use for the LCA study. Since we have described that the use of FIL affects the impacts of the life cycle of the R-32 recovery, a replacement after one year, 6 months, or 1 month of use was considered. The relative comparison of the life cycle impacts of R-32 recovery in these three scenarios is compared with the benchmark scenario (R-32 production) in Figure 6 (environmental impacts of the benchmark scenario were considered 100%).

The comparison indicates that the periodicity of replacement of the FIL is not a critical factor in the sustainability of the circular economy approach. The ADP (elements) category is highly affected by replacing the FIL in a shorter time, reaching a similar impact than in the benchmark scenario if the FIL is replaced monthly. Additionally, HTP can reach 45% of the impact of the benchmark scenario, while the rest of the impact categories are below 24%, even if the FIL replacement is carried out monthly. According to these findings, the environmental impacts of R-32 recovery are slightly influenced by the FIL replacement time; a monthly period increases the environmental loads but still falls short of the environmental burdens of R-32 production, even in this very conservative scenario, in the majority of the impact categories.

## Conclusions

In this work, a methodology based on the COSMO-RS thermodynamic package integrated into an Aspen Plus process simulator was used to evaluate the performance of the FIL [C_2_mim][C_4_F_9_CO_2_] to recover R-32 from the commercial mixture R-407F. Results from the absorption simulation reveal that it is possible to recover 30.3% of 98% weight purity R-32 suitable for further reuse with an absorption column of 38 theoretical stages and a working pressure of 8 bar.

The sustainability of the R-32 recovery process (circular economy scenario) was assessed with an LCA methodology. The obtained results were compared with the environmental impacts of the conventional R-32 production (benchmark scenario). The LCA results show that the R-32 recovery from wasted R-407F has considerably lower environmental impacts than the production of fresh R-32, with a GWP reduction of 86%. Moreover, the results evidence that the FIL production and the fugitive emissions in the FIL use stage are the main hotspots dominating the life cycle of the R-32 recovery scenario. The production stage of FIL contributes more than 57% to most of the impact categories, while the fugitive emissions are the main ones responsible for the impact in the GWP category.

Sensitivity analysis results show that decreasing the time of FIL replacement has a significant impact in the ADP (elements) and HTP categories, but still, the impacts are generally lower than in the R-32 production scenario in most of the categories.

In conclusion, the LCA results show that developing new recovery technologies for F-gases in a circular economy context has a high potential for reducing its environmental impact.

## Supporting Information Available

Computational details and COSMO-RS implementation; impact categories; sigma profile for the [C_2_mim][C_4_F_9_CO_2_] molecule; complete process scheme used in Aspen Plus v11; characterization of the environmental impacts of conventional production of 1 kg of R-32; distribution of the normalized environmental impacts of (A) conventional production of 1 kg of R-32 and (B) recovery of 1 kg of R-32 from R-407F with [C_2_mim][C_4_F_9_CO_2_]; uncertainty analysis; scalar properties of [C_2_mim][C_4_F_9_CO_2_]; adjusted temperature-dependent properties for [C_2_mim][C_4_F_9_CO_2_]; the LCI data for the production of 1 kg of the [C_2_mim][C_4_F_9_CO_2_]; the LCI results of production of 1 kg of [C_2_mim][C_4_F_9_CO_2_]; the LCI data for the recovery of 1 kg of R-32 from R-407F; and characterization of the environmental impacts of conventional production of 1 kg of R-32 (PDF)
