# Supplementary material for: Life Cycle Assessment of the Separation and Recycling of Fluorinated Gases Using Ionic Liquids in a Circular Economy Framework
Source: ACS Sustain Chem Eng. 2021 Dec 17;10(1):71–80. doi: 10.1021/acssuschemeng.1c04723 (PMC8753992; doi:10.1021/acssuschemeng.1c04723)
Supplement: Supplementary file 1 — sc1c04723_si_001.pdf [file sc1c04723_si_001.pdf]

SUPPORTING INFORMATION

Life Cycle Assessment of the Separation and Recycling of Fluorinated Gases using Ionic Liquids in a Circular Economy Framework

Daniel Jovell[†]*, Josep O. Pou*[†]*, Fèlix Llovell*[‡]*, Rafael Gonzalez-Olmos*[†*]

† Department of Chemical Engineering and Materials Science, IQS School of Engineering, Universitat Ramon Llull, Via Augusta 390, 08017 Barcelona, Spain.

‡ Department of Chemical Engineering, Universitat Rovira i Virgili, Avinguda Països Catalans 26, 43007, Tarragona, Spain.

***Corresponding Author:** E-mail: rafael.gonzalez@iqs.url.edu

Contents: 23 pages, 5 figures, and 7 tables

A1. Computational Details and COSMO-RS implementation

COSMO-SAC is implemented in the model of Lin and Sandler[1] as a liquid activity coefficient model, although two other different versions are available using the option codes in the Aspen Plus interface: the COSMO-RS (conductor-like screening model for real solvents) model by Klamt and Eckert and the modification in the exchange energy of the original Lin and Sandler model.[2] The original COSMO-RS model has been used in this contribution as other authors recommend[3] since it presents the lowest deviations from experimental data. The liquid activity coefficient is obtained as:

$$\ln \gamma_i = \frac{A_i}{a_{eff}} \sum_{\sigma_m} p_i(\sigma_m)[\ln \Gamma_s(\sigma_m) - \ln \Gamma_i(\sigma_m)] + \ln \gamma_i^{SG} \quad (1)$$

Where A_i is the molecular surface of component i, a_{eff} is the standard segment surface area (7.50 Å^2), $p_i(\sigma_m)$ is the sigma profile of component i, $\Gamma_s(\sigma_m)$ is the segment activity coefficient of segment σ_m in the component i, and γ_i^{SG} Is the Staverman-Guggenheim model for combinatorial contribution to γ_i.

COSMO-RS calculations for each independent cation-anion conforming the FIL are performed. When available, the considered species were added from the COSMObase libraries. For the anion and cation, optimized structures have been generated with TmoleX software v.21.0.1[4] using the triple-ζ valence potential (def-TZVP) basis with the Becke and Perdew (BP) functional at the density functional theory (DFT) level using the resolution of identity (RI) approximation and a convergence criterion of 10^{-6} Hartree, following the recommendations of other authors.[3,5,6] COSMO files were then generated from the optimized structures by applying the COSMO solvation model at the same quantum-chemical computational level. The files were used as an input in COSMOThermX software v.21.0[7] and its parametrization BP_TZVP_C30_1701 to perform COSMO-RS calculations to obtain the molecular volume and σ-profiles required

by the fluid package. It should be noted that the σ-profile for the FIL considered are obtained as the sum of the cation and anion σ-profiles. The obtained σ-profile is presented in Figure S1 and Table S1 from Supporting Information. Palomar and co-workers have widely used this approach; the reader is referred to their publications[3,8–11] for further insight on the methodology.

A2. Impact Categories

- Global warming: The characterization model as developed by the Intergovernmental Panel on Climate Change (IPCC) is selected to develop characterization factors. Factors are expressed as global warming potential (GWP) for time horizon 100 years (GWP100), in kg CO_2 eq./kg emission.
- Depletion of abiotic resources: comprises two impact categories, abiotic depletion of elements (ADP elements, ultimate reserves) and abiotic depletion of fossil fuels (ADP fossil fuels). ADP element is related to the extraction of minerals due to inputs in the system. The abiotic depletion factor (ADF) is determined for each extraction of minerals (kg antimony eq./kg extraction) based on concentration reserves and the rate of deaccumulation.
- ADP fossil fuels are related to the lower heating value (LHV) expressed in MJ per kg of m^3 fossil fuel. The reason for taking the LHV is that fossil fuels are considered to be fully substitutable.
- Ozone layer depletion: The characterization model was developed by the World Meteorological Organization (WMO) and defines ozone depletion potential (ODP) of different gases (kg CFC-11 eq./kg emission).
- Human toxicity: expressed as human toxicity potential (HTP). It can be calculated using USES-LCA to describe fate, exposure, and effects of toxic substances for an infinite time horizon. For each toxic substance, HTP is expressed as kg 1,4-dichlorobenzene eq./kg emission.
- Freshwater aquatic ecotoxicity: expressed as freshwater aquatic ecotoxicity potential (FAETP). The unit is kg 1,4-dichlorobenzene eq./kg emission. See description human toxicity.

- Marine aquatic ecotoxicology: expressed as marine aquatic ecotoxicology potential (MAETP). The unit is kg 1,4-dichlorobenzene eq./kg emission. See description human toxicity.
- Terrestrial ecotoxicity: expressed as terrestrial ecotoxicity potential (TETP). The unit is kg 1,4-dichlorobenzene eq./kg emission. See description human toxicity.
- Photochemical oxidation: Photochemical Ozone Creation Potential (POCP) for emission of substances to air is calculated with the UNECE Trajectory model (including fate), and is expressed as kg ethylene eq. per kg emission.
- Acidification: Acidification Potential (AP) for emissions to air is calculated with the adapted RAINS 10 model, describing the fate and deposition of acidifying substances. AP is expressed as acidification potential (AP) with the unit kg SO_2 eq. per kg emission.
- Eutrophication: Eutrophication potential (EP) is based on the stoichiometric procedure of Heijungs (1992). EP is expressed with the unit kg PO_4 eq. per kg emission.

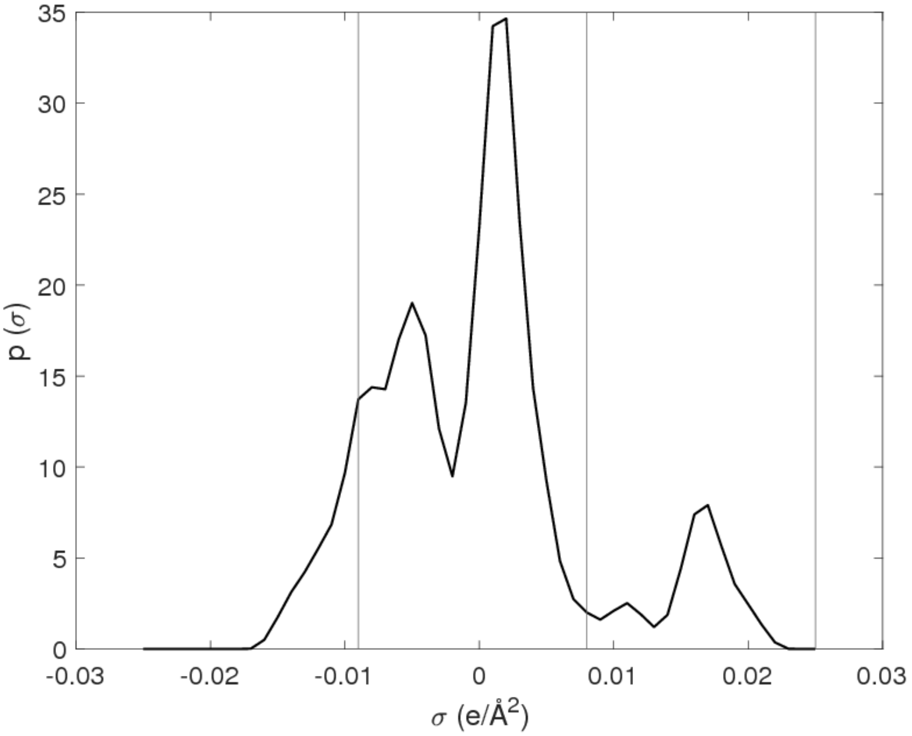

Figure S1. Sigma profile for the $[C_2mim][C_4F_9CO_2]$ molecule

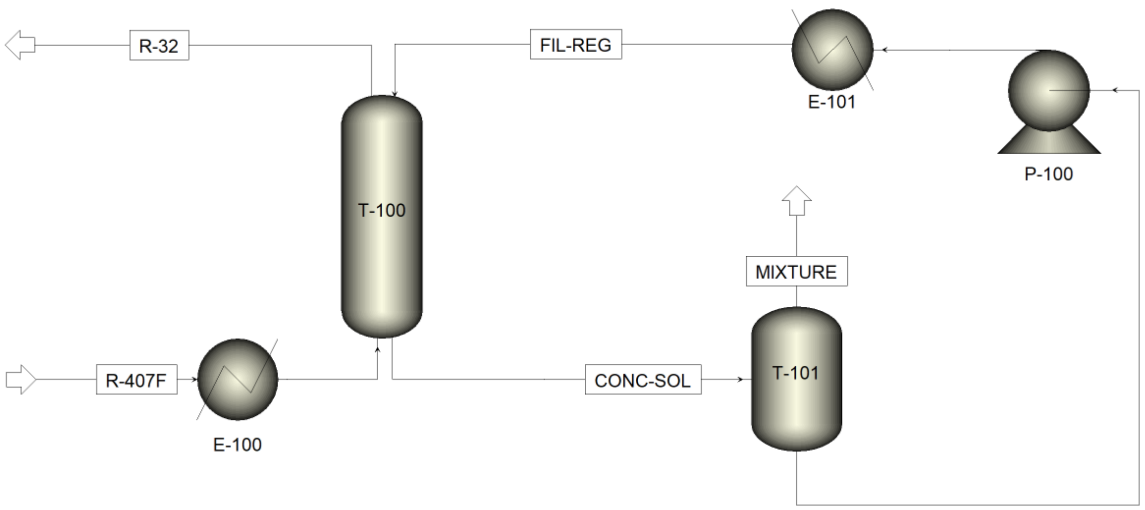

Figure S2. Complete Process Scheme Used in Aspen Plus v11

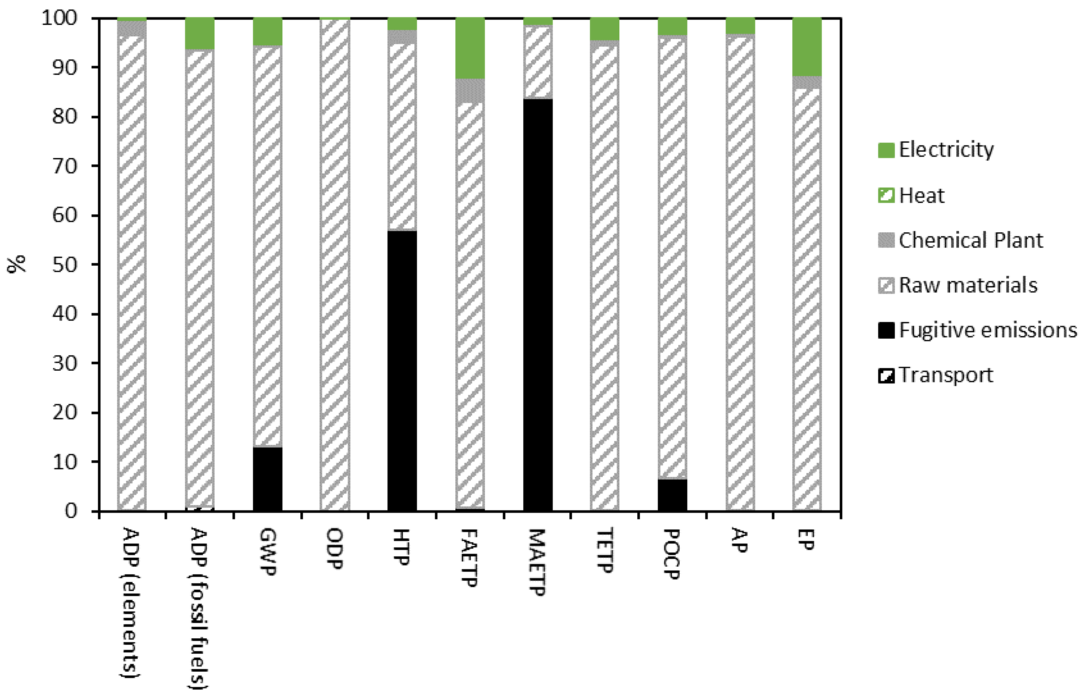

Figure S3. Characterization of the environmental impacts of conventional production of 1 kg of R-32.

A

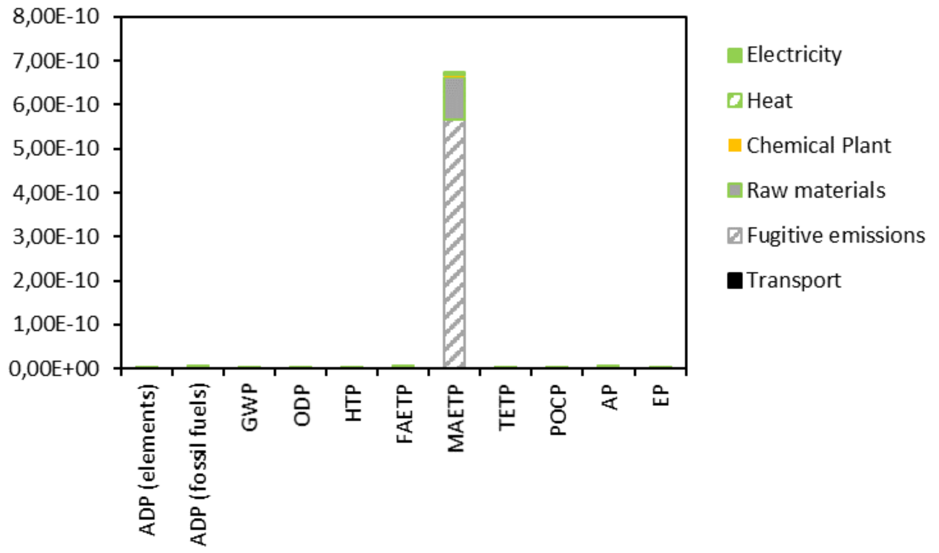

B

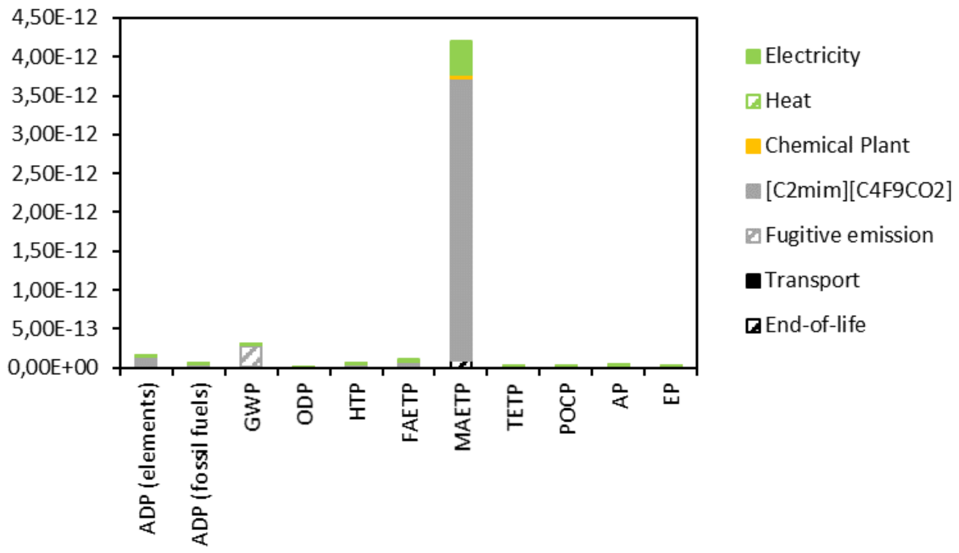

Figure S4. Distribution of the normalized environmental impacts of A) conventional production of 1 kg of R-32 and B) recovery of 1 kg of R-32 from R-407F with [C_2mim][$C_4F_9CO_2$].

A3. Uncertainty Analysis

The uncertainty analysis for the two scenarios studied (R-32 production and recovery) was conducted to test the confidence level of the results reported in this work. The Monte Carlo simulation (with 1000 iterations) results are presented in Figure S5. The results show that in all categories, the R-32 recovery scenario has a lower environmental load than the R-32 production scenario, which means it is certain that the recovery of R-32 with FIL is more environmentally benign than its new production. The results can give further confirmation on the R-32 recovery LCA results reported in this work from a statistical aspect.

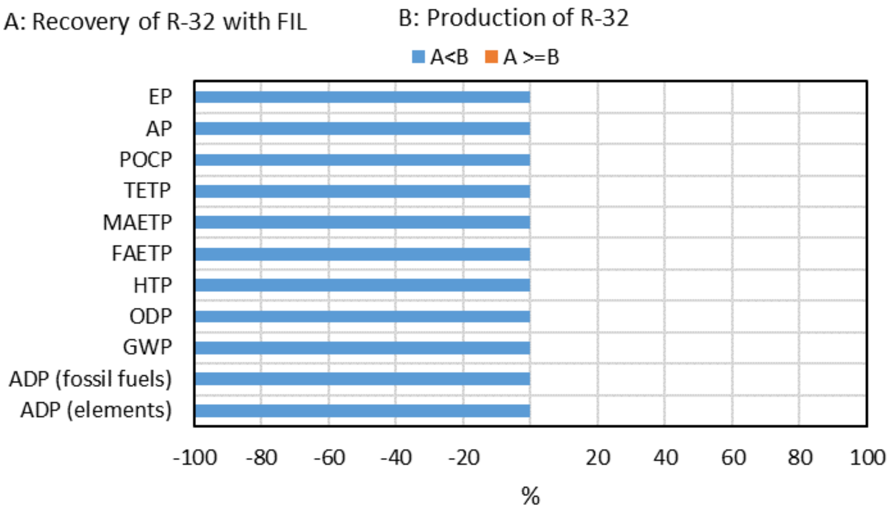

Figure S5. Uncertainty analysis for the recovery of R-32 with FIL (A) and comparison with R-32 production (B) (functional unit: 1 kg of R-32)

Table S1. Sigma profile for the $[C_2mim][C_4F_9CO_2]$ molecule

**SGPRF-1**											
1	2	3	4	5	6	7	8	9	10	11	12
0	0	0	0	0	0	0	0	0.017	0.502	1.753	3.119
**SGPRF-2**											
1	2	3	4	5	6	7	8	9	10	11	12
4.22	5.5	6.838	9.682	13.723	14.384	14.278	17.011	19.015	17.24	12.104	9.493
**SGPRF-3**											
1	2	3	4	5	6	7	8	9	10	11	12
13.543	23.209	34.235	34.652	23.48	14.346	9.21	4.834	2.743	2.003	1.611	2.098
**SGPRF-4**											
1	2	3	4	5	6	7	8	9	10	11	12
2.518	1.913	1.207	1.873	4.435	7.394	7.907	5.682	3.57	2.462	1.342	0.36
**SGPRF-5**											
1	2	3									
0.011	0	0									

Table S2 Scalar properties for $[C_2mim][C_4F_9CO_2]$

Property	Value
Molecular weight, g/mol	374.20
Normal boiling point , K	627.97
Density at 60ºF, g/cm^3	1.49616
Critical temperature, K	814.44
Critical pressure, bar	15.285
Critical volume, cm^3/mol	847.56
Critical compressibility factor	0.19131
Pitzer acentric factor	0.71376
COSMO Volume, $Å^3$	365.448

Table S3 Adjusted temperature-dependent properties for [C_2mim][$C_4F_9CO_2$]

Parameter	Physical Property	Property Units	C1	C2	C3	C4	C5	T range (K)
CPLPO	Heat Capacity	J/(kmol·k)	566.7	41.14	-1.729	-4.116E-4	-5.997E-3	250K - 550
DNLDIP	Liquid density	mol/cm^3	4.694E-4	0.3119	1130	0.5964		250 - 500
MULDIP	Liquid viscosity	cP	-217.1	13830	30.78			250 - 500
PLXANT	Vapor Pressure	bar	-1E+18					200-600

Table S4 The LCI data for the production of 1 kg of the [C_2mim][$C_4F_9CO_2$]

Inventory	Quantity	Unit	Data Source
**Input**			Vieira et al.[12] and simulation work
[C_2mim]Br	0.511	kg	
C_4F_9COOH	0.706	kg	
Water	1.411	kg	
Heat	6.630	MJ_{th}	
Electric Energy	0.120	MJ_e	
**Output**			
[C_2mim][$C_4F_9CO_2$]	1	kg	
Hydrogen bromide	0.216	kg	
Water	1.411	kg	
**Input**			Liu, Z.;[13] Wu et al.[14]
N-methylimidazole	0.224	kg	
Bromoethane	0.357	kg	
Heat	5.57	MJ_{th}	
Electric Energy	0.594	MJ_e	
**Output**			
[C_2mim]Br	0.511	kg	
**Input**			Righi et al.;[15] Wu et al.[14]
Glyoxal	0.531	kg	
Methylamine	0.114	kg	
Formaldehyde	0.297	kg	
Ammonia	0.064	kg	
Heat	1.40	MJ_{th}	
Electric Energy	0.700	MJ_e	
**Output**			
N-methylimidazole	0.224	kg	
Other	0.782	kg	
**Input**			Mehrkesh et al.;[16] Wu et al.[14]

Ethanol	0.154	kg	
Hydrobromic Acid	0.273	kg	
Heat	1.12	MJ_{th}	
Electric Energy	0.254	MJ_e	
**Output**			
Bromoethane	0.357	kg	
Water	0.070	kg	
**Input**			Knauck et al.[17] and simulation work
Pentanoyl chloride	0.322	kg	
Hydrogen fluoride	0.535	kg	
Water	0,048		
Heat		MJ_{th}	
Electric Energy	11.596	MJ_e	
**Output**			
C_4F_9COOH	0.706	kg	
Water	0.070	kg	
Hydrogen fluoride	0.054	kg	
Hydrogen	0.049	kg	
Hydrogen chloride	0.097	kg	
**Input**			Larock et al.[18] and simulation work
Pentanoic acid	0.273	kg	
Thionyl chloride	0.318	kg	
Heat		MJ_{th}	
Electric Energy	2.853	MJ_e	
**Output**			
Pentanoyl chloride	0.322	kg	
Sulphur dioxide	0.171	kg	
Hydrogen chloride	0.097	kg	
**Input**			Larock et al.[18] and simulation work
Pentanol	0.236	kg	

Potassium dichromate	0.524	kg
Heat		MJ_{th}
Electric Energy	2.156	MJ_e
**Output**		
Pentanoic acid	0.273	kg
Chromium oxide	0.271	kg
Potassium oxide	0.168	kg
Water	0.048	kg

Table S5. Life cycle impact results of production of 1 kg of [C_2mim][$C_4F_9CO_2$]

**Impact category**	**Units**	**Total**	**Fugitive emissions**	**[C_2mim]Br**	**C_4F_9COOH**	**Heat**	**Electricity**	**Transport**
ADP (elements)	kg Sb eq.	$1.45 \cdot 10^{-3}$	0	$1.21 \cdot 10^{-3}$	$2.40 \cdot 10^{-4}$	$8.33 \cdot 10^{-8}$	$1.05 \cdot 10^{-7}$	$1.44 \cdot 10^{-7}$
ADP (fossil fuels)	MJ	$1.74 \cdot 10^{2}$	0	$7.45 \cdot 10^{1}$	78.18	$1.84 \cdot 10^{1}$	2.63	$8.36 \cdot 10^{-1}$
GWP	kg CO_2 eq.	9.99	0	3.38	5.31	1.08	$2.11 \cdot 10^{-1}$	$5.24 \cdot 10^{-2}$
ODP	kg CFC-11 eq.	$1.37 \cdot 10^{-6}$	0	$4.44 \cdot 10^{-7}$	$7.34 \cdot 10^{-7}$	$1.61 \cdot 10^{-7}$	$2.86 \cdot 10^{-8}$	$9.90 \cdot 10^{-9}$
HTP	kg 1,4-DB eq.	$4.29 \cdot 10^{1}$	$4.94 \cdot 10^{-4}$	$3.64 \cdot 10^{1}$	6.38	$5.75 \cdot 10^{-2}$	$5.45 \cdot 10^{-2}$	$1.69 \cdot 10^{-2}$
FAETP	kg 1,4-DB eq.	4.56	$4.91 \cdot 10^{-3}$	$7.51 \cdot 10^{-1}$	3.72	$3.91 \cdot 10^{-2}$	$4.68 \cdot 10^{-2}$	$4.46 \cdot 10^{-3}$
MAETP	kg 1,4-DB eq.	$5.28 \cdot 10^{4}$	$9.68 \cdot 10^{-4}$	$2.65 \cdot 10^{3}$	$4.98 \cdot 10^{4}$	$1.30 \cdot 10^{2}$	$2.53 \cdot 10^{2}$	$1.46 \cdot 10^{1}$
TETP	kg 1,4-DB eq.	$1.77 \cdot 10^{-2}$	$5.58 \cdot 10^{-4}$	$4.98 \cdot 10^{-3}$	$1.13 \cdot 10^{-2}$	$3.46 \cdot 10^{-4}$	$5.67 \cdot 10^{-4}$	$7.26 \cdot 10^{-5}$
POCP	kg C_2H_4 eq.	$4.08 \cdot 10^{-3}$	$4.31 \cdot 10^{-4}$	$1.10 \cdot 10^{-3}$	$2.40 \cdot 10^{-3}$	$9.80 \cdot 10^{-5}$	$5.75 \cdot 10^{-5}$	$8.55 \cdot 10^{-6}$
AP	kg SO_2 eq.	$6.80 \cdot 10^{-2}$	0	$1.52 \cdot 10^{-2}$	$5.00 \cdot 10^{-2}$	$1.28 \cdot 10^{-3}$	$1.44 \cdot 10^{-3}$	$1.69 \cdot 10^{-4}$
EP	kg PO_4 eq.	$1.45 \cdot 10^{-2}$	0	$4.39 \cdot 10^{-3}$	$9.59 \cdot 10^{-3}$	$2.61 \cdot 10^{-4}$	$2.66 \cdot 10^{-4}$	$3.72 \cdot 10^{-5}$

Table S6. The LCI data for the recovery of 1 kg of R-32 from R-407F

Inventory	Quantity	Unit	Data Source
**Input**			Simulation work
$[C_2mim][C_4F_9CO_2]$	0.008	kg	
R-407F	11.00	kg	
Heat	0.184	MJ_{th}	
Electric Energy	0.340	MJ_e	
**Output**			
R-32	1.00	kg	
$[C_2mim][C_4F_9CO_2]$	0.008	kg	
Mixture R-32/R-134a/R-125	10.00	kg	

Table S7. The LCI data for the production of 1 kg of R-32

Inventory	Quantity	Unit	Data Source
**Input**			Yuichi et al.;[19] Ecoinvent HFC-152 production and Simulation work
Hydrogen Fluoride	0.81	kg	
Dichloromethane	1.72	kg	
Chromium	0.002	kg	
Heat	0.931	MJ_{th}	
Electric Energy	3.920	MJ_e	
**Output**			
R-32	1	kg	
Hydrogen chloride	1.400	kg	

REFERENCES

(1) Lin, S.-T.; Sandler, S. I. A Priori Phase Equilibrium Prediction from a Segment Contribution Solvation Model. *Ind. Eng. Chem. Res.* **2002**, *41* (5), 899–913. https://doi.org/10.1021/ie001047w.

(2) Lin, S. T.; Mathias, P. M.; Song, Y.; Chen, C. C.; Sandler, S. I. Improvements of Phase-Equilibrium Predictions for Hydrogen-Bonding Systems from a New Expression for COSMO Solvation Models. In *AIChE Annual Meeting, Indianapolis, IN*; 2002; pp 3–8.

(3) Sosa, J. E.; Santiago, R.; Hospital-Benito, D.; Costa Gomes, M.; Araújo, J. M. M.; Pereiro, A. B.; Palomar, J. Process Evaluation of Fluorinated Ionic Liquids as F-Gas Absorbents. *Environ. Sci. Technol.* **2020**, *54* (19), 12784–12794. https://doi.org/10.1021/acs.est.0c05305.

(4) Balasubramani, S. G.; Chen, G. P.; Coriani, S.; Diedenhofen, M.; Frank, M. S.; Franzke, Y. J.; Furche, F.; Grotjahn, R.; Harding, M. E.; Hättig, C.; Hellweg, A.; Helmich-Paris, B.; Holzer, C.; Huniar, U.; Kaupp, M.; Marefat Khah, A.; Karbalaei Khani, S.; Müller, T.; Mack, F.; Nguyen, B. D.; Parker, S. M.; Perlt, E.; Rappoport, D.; Reiter, K.; Roy, S.; Rückert, M.; Schmitz, G.; Sierka, M.; Tapavicza, E.; Tew, D. P.; van Wüllen, C.; Voora, V. K.; Weigend, F.; Wodyński, A.; Yu, J. M. TURBOMOLE: Modular Program Suite for Ab Initio Quantum-Chemical and Condensed-Matter Simulations. *J. Chem. Phys.* **2020**, *152* (18), 184107. https://doi.org/10.1063/5.0004635.

(5) García-Gutiérrez, P.; Jacquemin, J.; McCrellis, C.; Dimitriou, I.; Taylor, S. F. R.; Hardacre, C.; Allen, R. W. K. Techno-Economic Feasibility of Selective CO2 Capture Processes from Biogas Streams Using Ionic Liquids as Physical

Absorbents. *Energy & Fuels* **2016**, *30* (6), 5052–5064. https://doi.org/10.1021/acs.energyfuels.6b00364.

(6) Aldawsari, J. N.; Adeyemi, I. A.; Bessadok-Jemai, A.; Ali, E.; AlNashef, I. M.; Hadj-Kali, M. K. Polyethylene Glycol-Based Deep Eutectic Solvents as a Novel Agent for Natural Gas Sweetening. *PLoS One* **2020**, *15* (9), e0239493. https://doi.org/10.1371/journal.pone.0239493.

(7) Dassault Systèmes. BIOVIA COSMOtherm http://www.3ds.com (accessed Nov 8, 2021).

(8) Ferro, V. R.; Ruiz, E.; De Riva, J.; Palomar, J. Introducing Process Simulation in Ionic Liquids Design/Selection for Separation Processes Based on Operational and Economic Criteria through the Example of Their Regeneration. *Sep. Purif. Technol.* **2012**, *97*, 195–204. https://doi.org/10.1016/j.seppur.2012.02.026.

(9) Larriba, M.; de Riva, J.; Navarro, P.; Moreno, D.; Delgado-Mellado, N.; García, J.; Ferro, V. R.; Rodríguez, F.; Palomar, J. COSMO-Based/Aspen Plus Process Simulation of the Aromatic Extraction from Pyrolysis Gasoline Using the {[4empy][NTf2] + [Emim][DCA]} Ionic Liquid Mixture. *Sep. Purif. Technol.* **2018**, *190*, 211–227. https://doi.org/10.1016/j.seppur.2017.08.062.

(10) Santiago, R.; Bedia, J.; Moreno, D.; Moya, C.; De Riva, J.; Larriba, M.; Palomar, J. Acetylene Absorption by Ionic Liquids: A Multiscale Analysis Based on Molecular and Process Simulation. *Sep. Purif. Technol.* **2018**, *204*, 38–48. https://doi.org/10.1016/j.seppur.2018.04.060.

(11) Abranches, D. O.; Larriba, M.; Silva, L. P.; Melle-Franco, M.; Palomar, J. F.; Pinho, S. P.; Coutinho, J. A. P. Using COSMO-RS to Design Choline Chloride

Pharmaceutical Eutectic Solvents. *Fluid Phase Equilib.* **2019**, *497*, 71–78. https://doi.org/10.1016/j.fluid.2019.06.005.

(12) Vieira, N. S. M.; Reis, P. M.; Shimizu, K.; Cortes, O. A.; Marrucho, I. M.; Araújo, J. M. M.; Esperança, J. M. S. S.; Lopes, J. N. C.; Pereiro, A. B.; Rebelo, L. P. N. A Thermophysical and Structural Characterization of Ionic Liquids with Alkyl and Perfluoroalkyl Side Chains. *RSC Adv.* **2015**, *5* (80), 65337–65350. https://doi.org/10.1039/c5ra13869h.

(13) Liu, Z. B. Method for Preparation of Imidazole Ionic Liquids by Microwave. CN108129391A2018.

(14) Wu, B.; Dai, C.; Chen, B.; Yu, G.; Liu, N.; Xu, R. Ionic Liquid versus Traditional Volatile Organic Solvent in the Natural Gas Dehydration Process: A Comparison from a Life Cycle Perspective. *ACS Sustain. Chem. Eng.* **2019**, *7* (23), 19194–19201.

(15) Righi, S.; Morfino, A.; Galletti, P.; Samorì, C.; Tugnoli, A.; Stramigioli, C. Comparative Cradle-to-Gate Life Cycle Assessments of Cellulose Dissolution with 1-Butyl-3-Methylimidazolium Chloride and N-Methyl-Morpholine-N-Oxide. *Green Chem.* **2011**, *13* (2), 367–375. https://doi.org/10.1039/C0GC00647E.

(16) Mehrkesh, A.; Karunanithi, A. T. Energetic Ionic Materials: How Green Are They? A Comparative Life Cycle Assessment Study. *ACS Sustain. Chem. Eng.* **2013**, *1* (4), 448–455. https://doi.org/10.1021/sc3001383.

(17) Kauck, E. A.; Diesslin, A. R. Some Properties of Perfluorocarboxylic Acids. *Ind. Eng. Chem.* **1951**, *43* (10), 2332–2334. https://doi.org/10.1021/ie50502a044.

(18) Larock, R. C. *Comprehensive Organic Transformations : A Guide to Functional Group Preparations*, 2nd ed.; Wiley-VCH: New York, 1999.

(19) Yuichi, I.; Ji, H.-S.; Cho, O.-J. Method of Producing Difluoromethane. US20040102659A1, 2002.
